# Supplementary figures and images for: Genome-wide identification and functional analysis of ARF transcription factors in Brassica juncea var. tumida
Source: PLoS One. 2020 Apr 22;15(4):e0232039. doi: 10.1371/journal.pone.0232039 (PMC7176091; doi:10.1371/journal.pone.0232039)

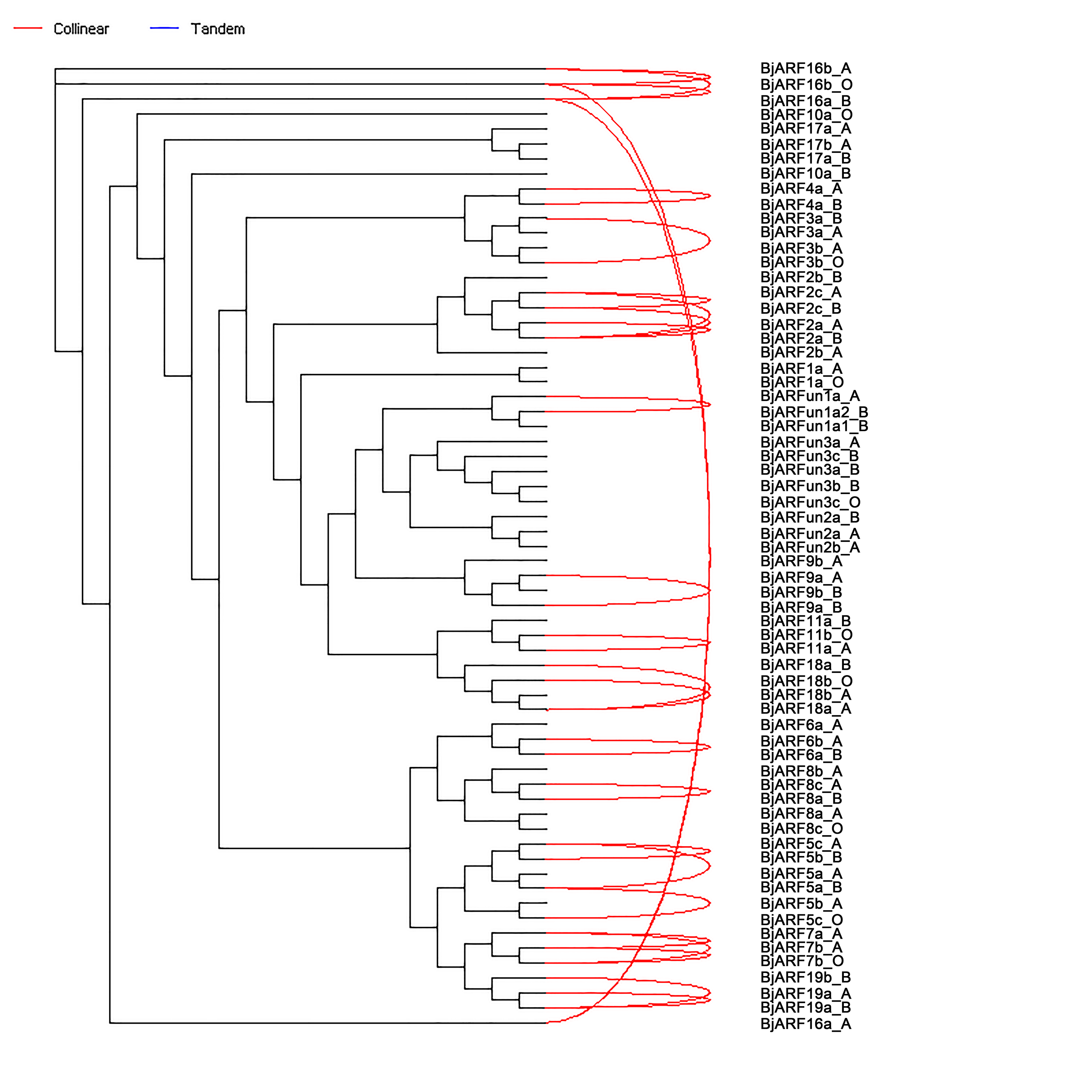

Supplement: S1 Fig — Curves connecting pairs of gene names suggest the collinear relationship. This annotated tree is output from ‘family tree plotter’ of MCscanX software. (PNG) [file pone.0232039.s006.png]

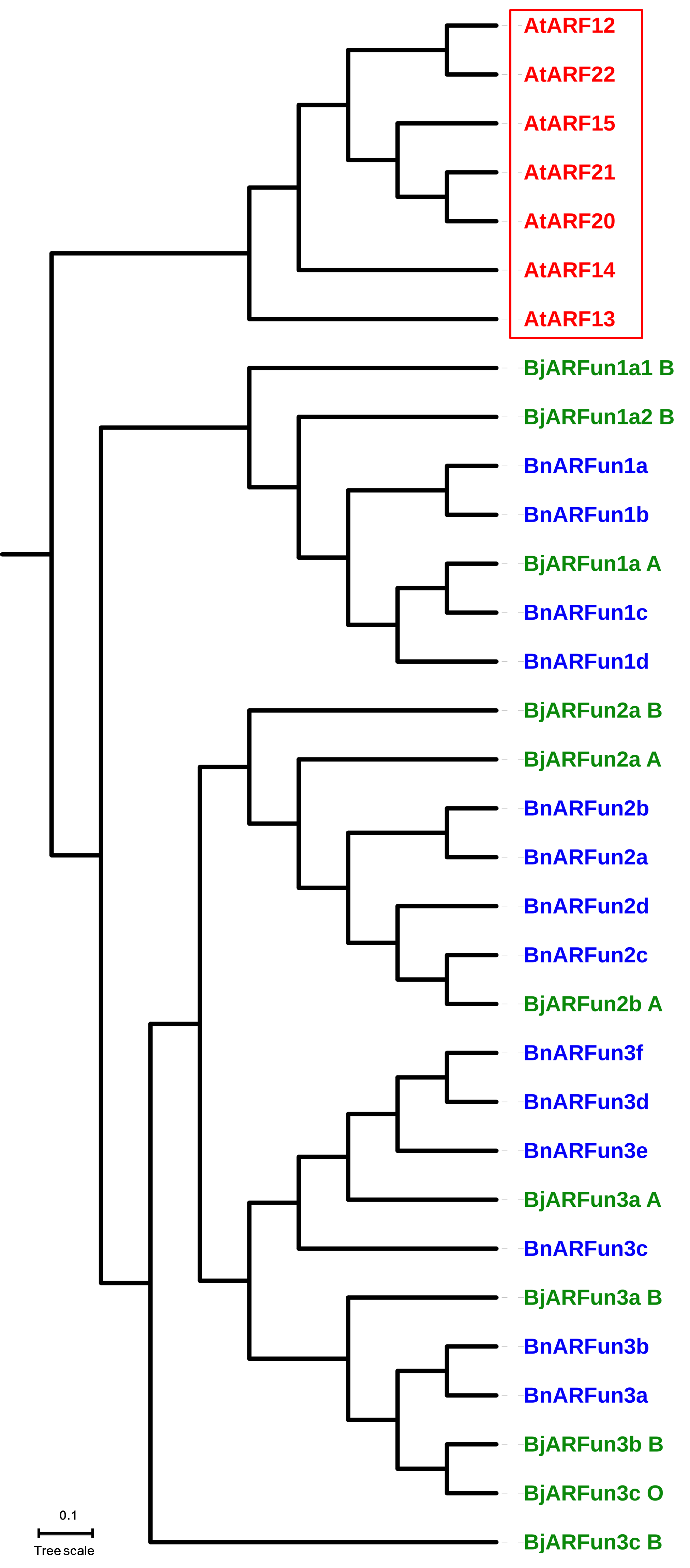

Supplement: S2 Fig — The phylogenetic tree was constructed using MEGA 7.0, the maximum likelihood method with 1,000 bootstraps. The different special of the ARF family are represented in different colors. (PNG) [file pone.0232039.s007.png]

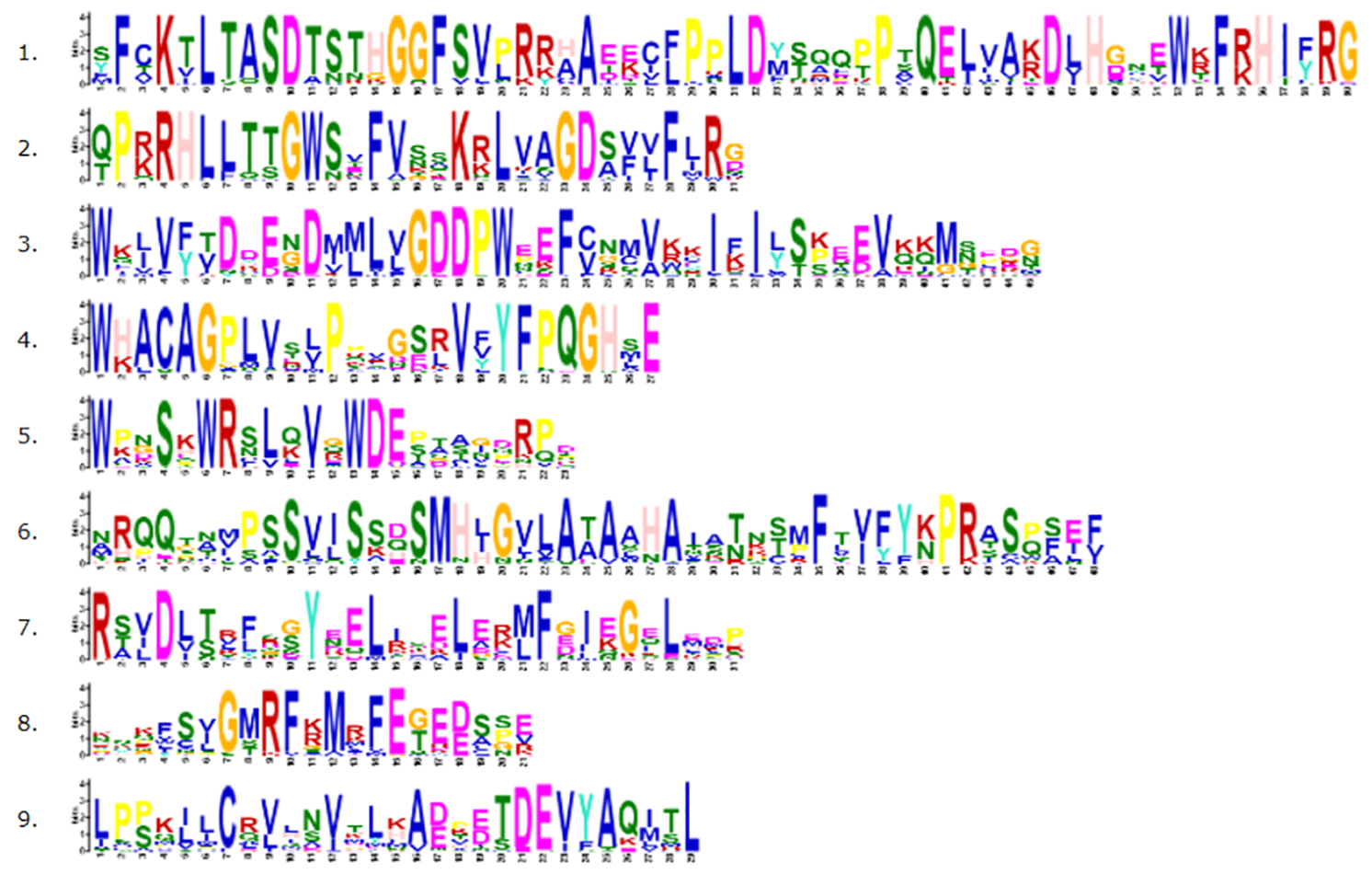

Supplement: S3 Fig — (PNG) [file pone.0232039.s008.png]

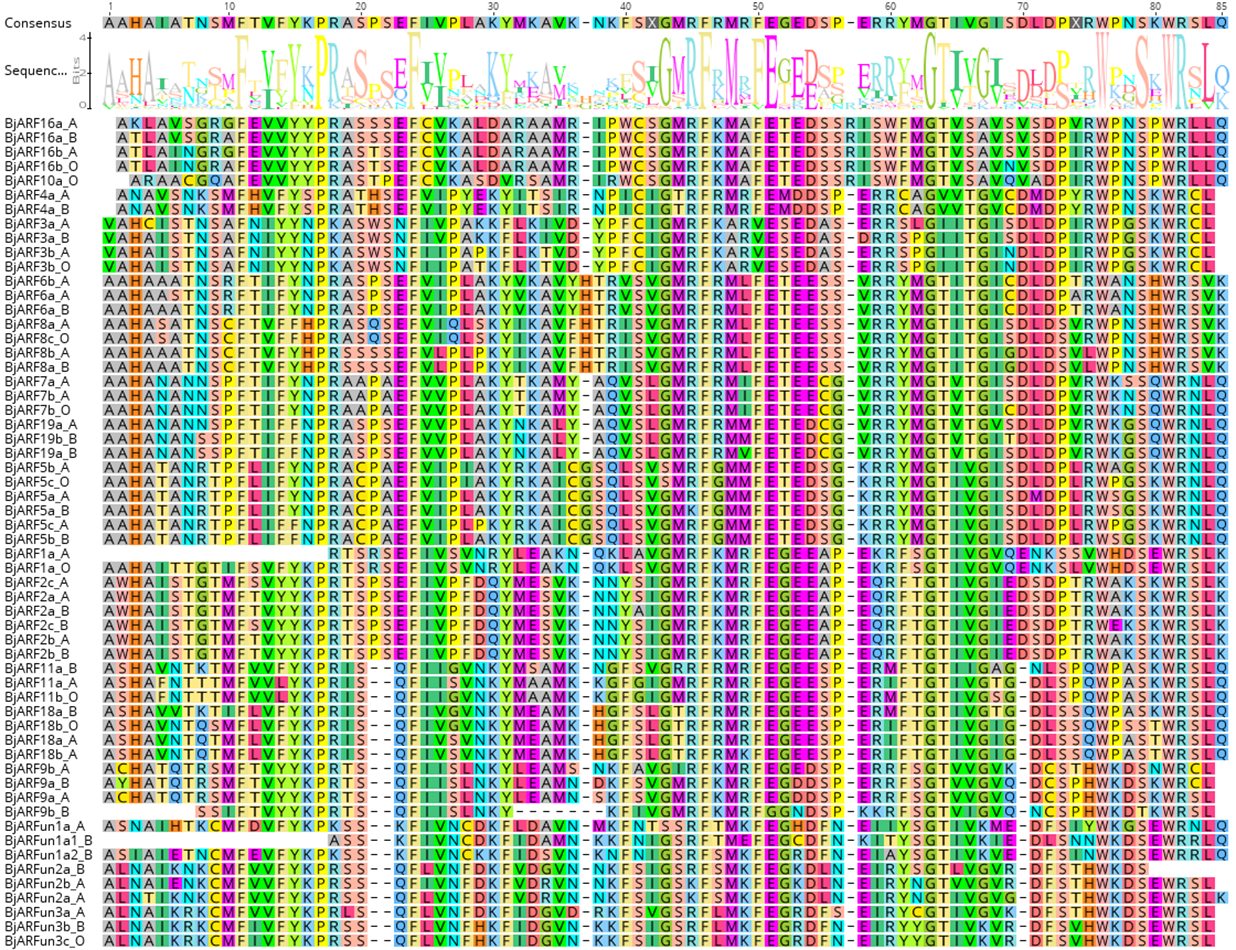

Supplement: S4 Fig — Multiple sequence alignment was performed using MAFFT version 7. (PNG) [file pone.0232039.s009.png]
